# Supplementary figures and images for: Temperature-Dependent Structural Variability of Prion Protein Amyloid Fibrils
Source: Int J Mol Sci. 2021 May 11;22(10):5075. doi: 10.3390/ijms22105075 (PMC8151363; doi:10.3390/ijms22105075)

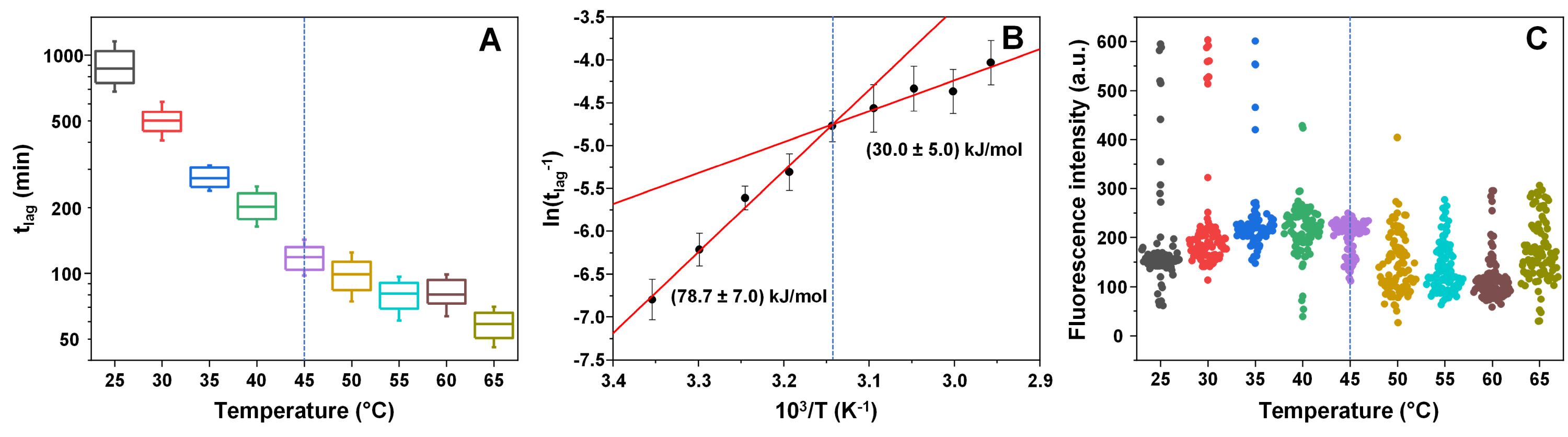

Supplement: Supplementary file 1 [file ijms-22-05075-s001.zip › Figure 1.tif]

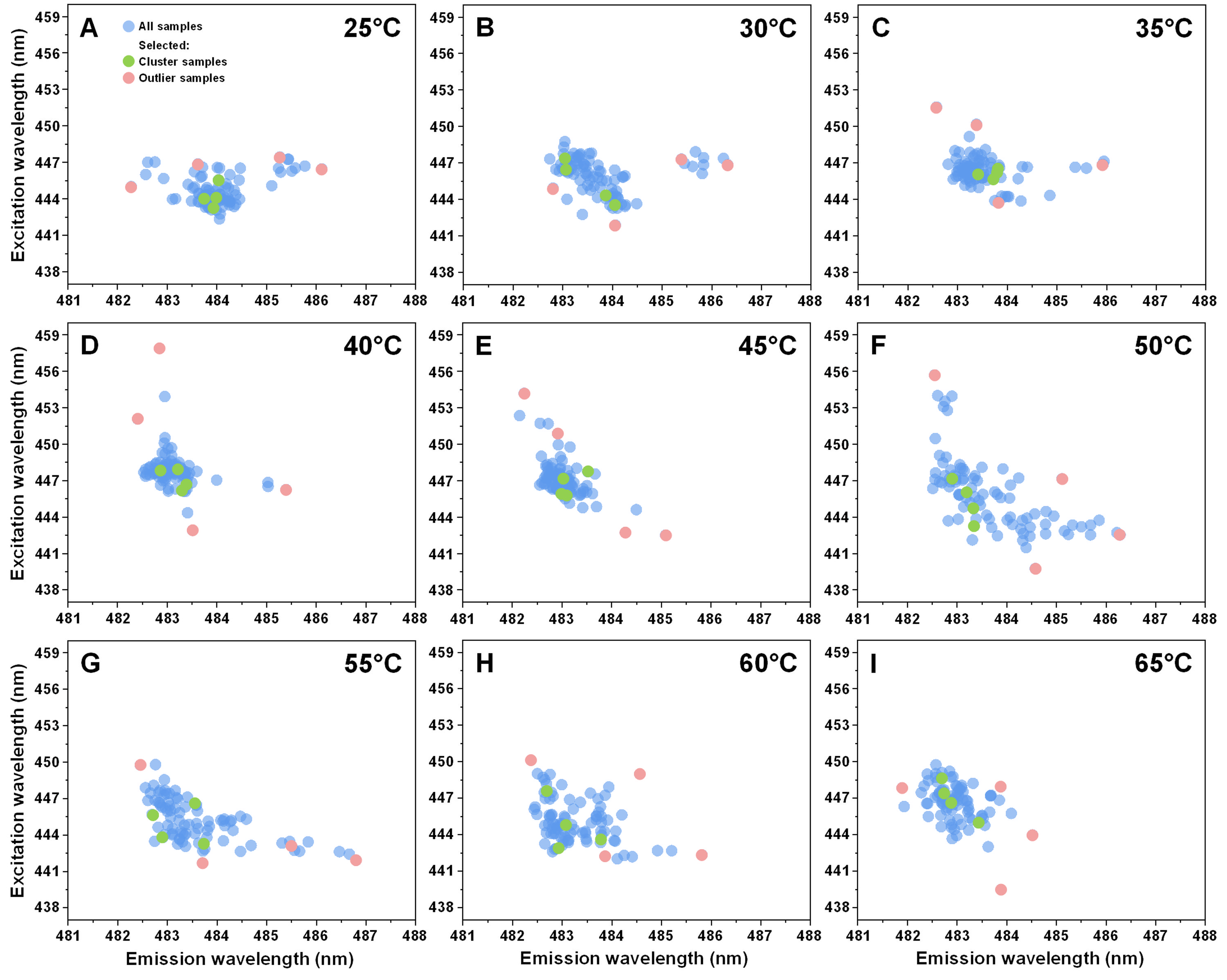

Supplement: Supplementary file 1 [file ijms-22-05075-s001.zip › Figure 2.tif]

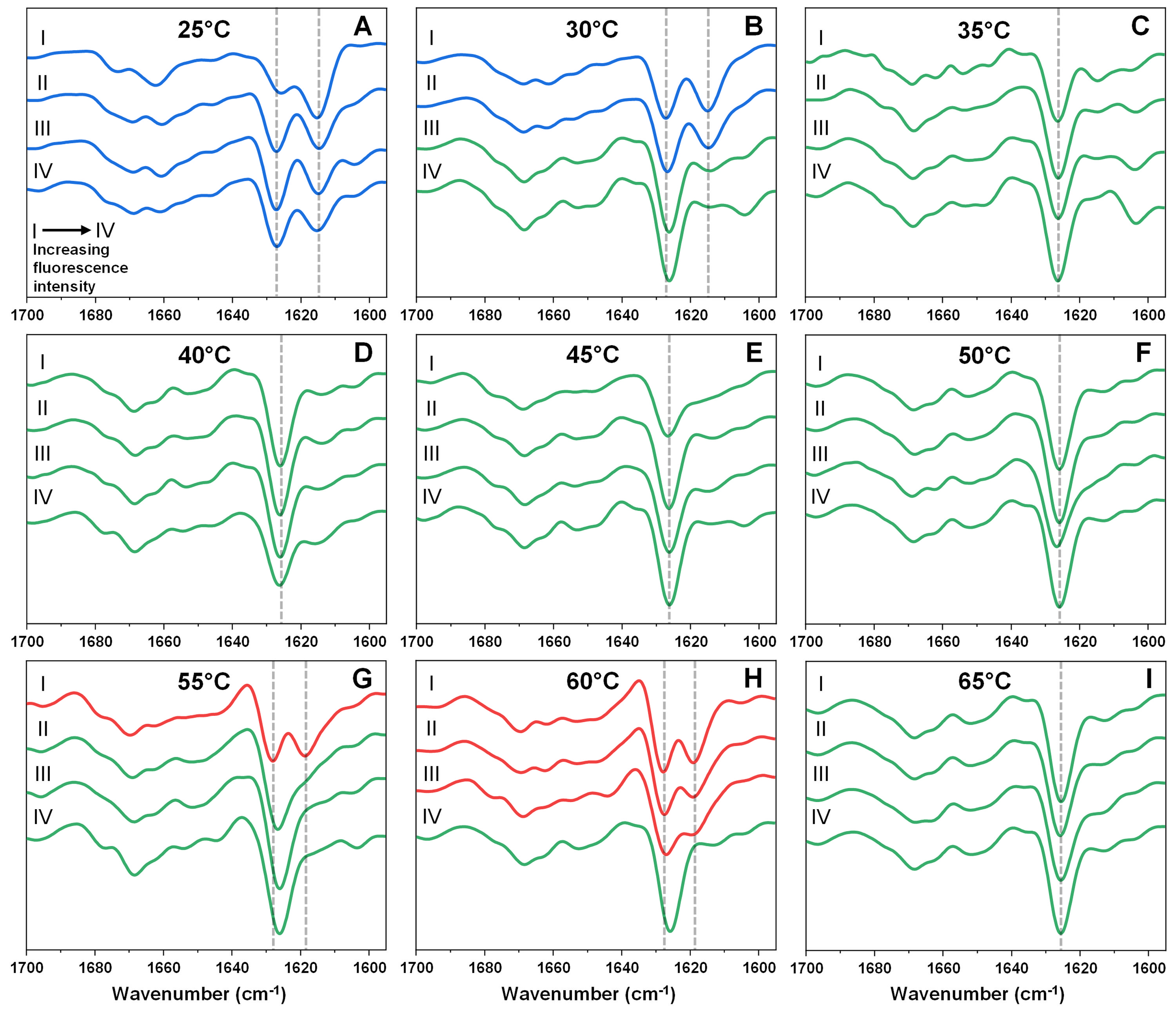

Supplement: Supplementary file 1 [file ijms-22-05075-s001.zip › Figure 3.tif]

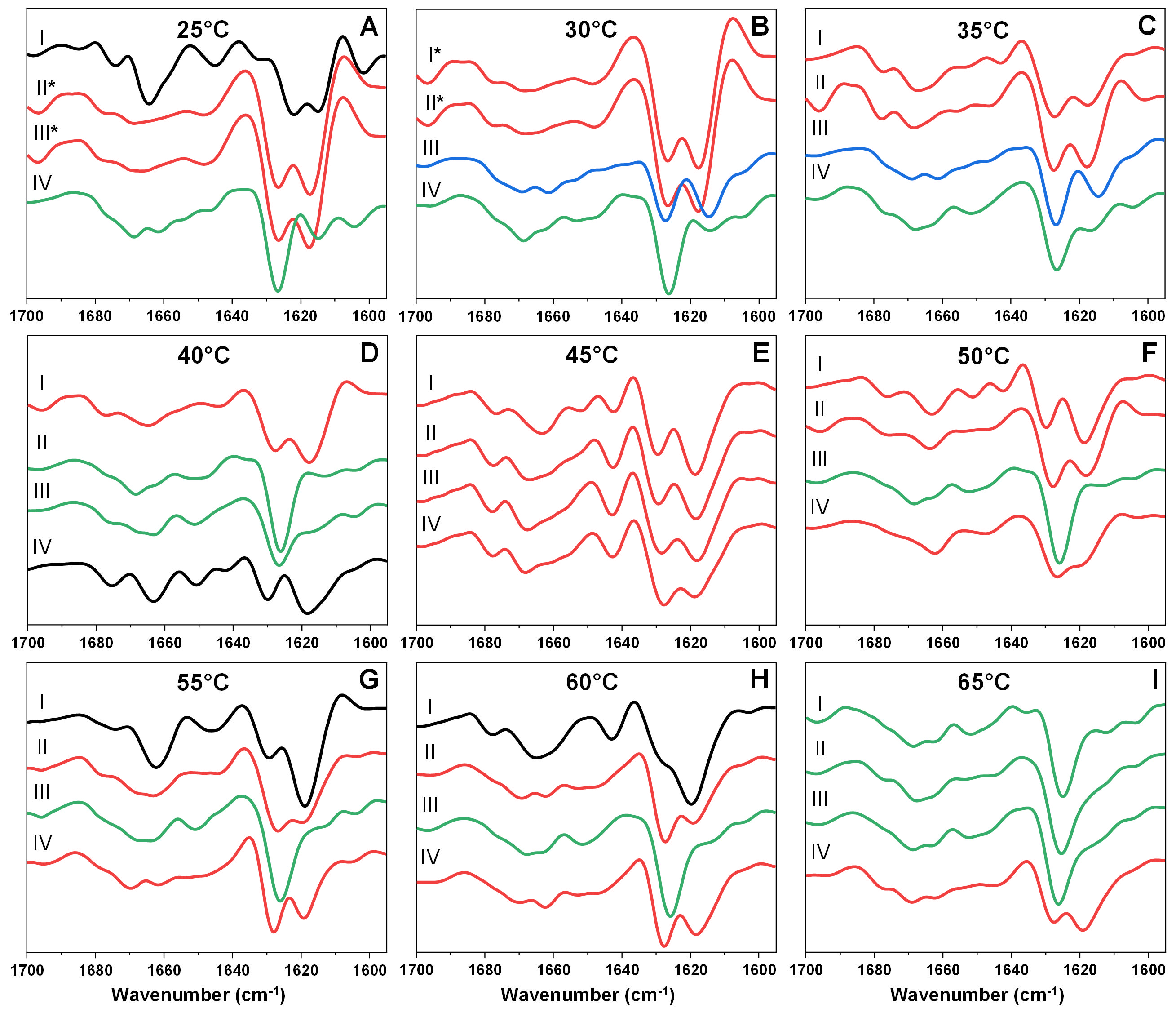

Supplement: Supplementary file 1 [file ijms-22-05075-s001.zip › Figure 4.tif]

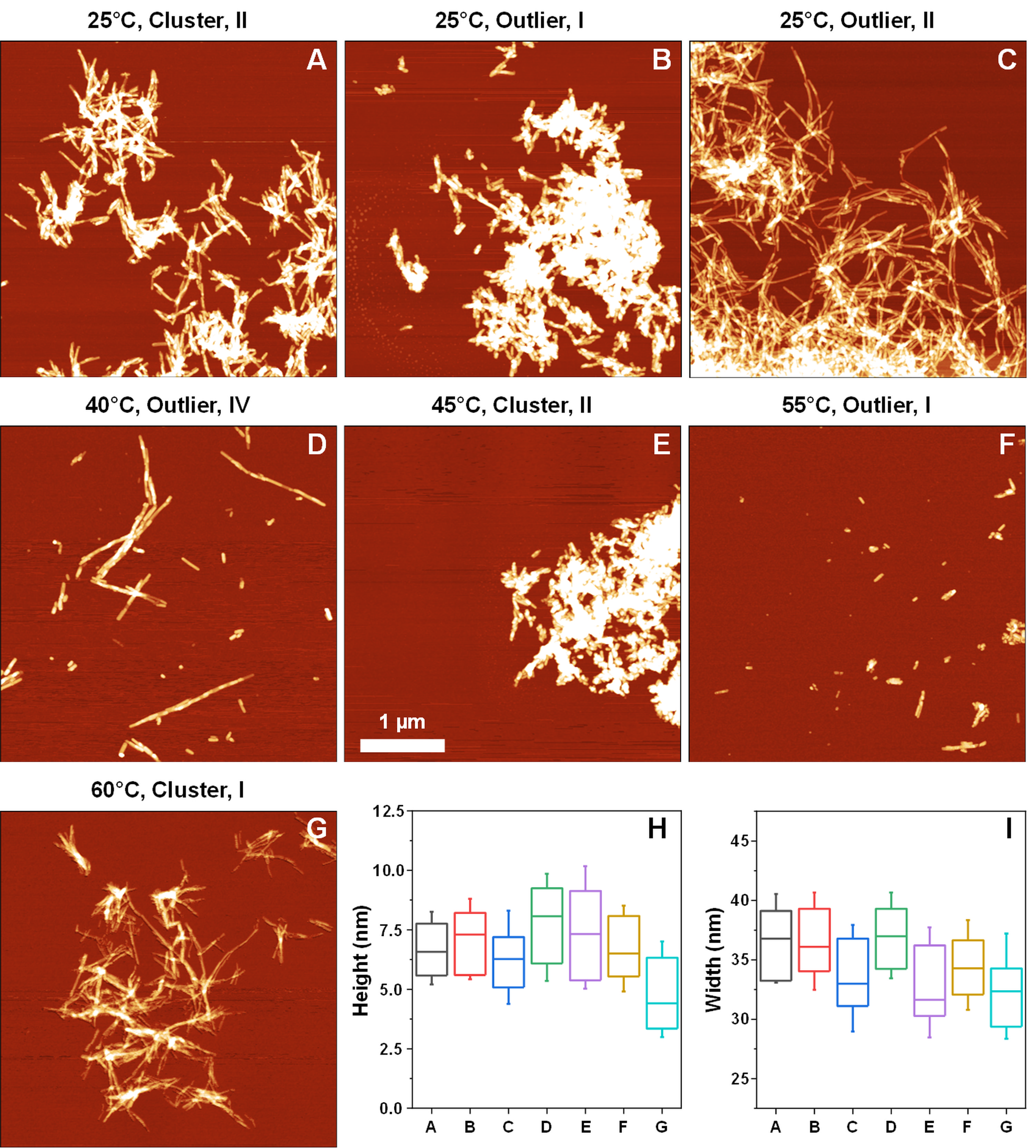

Supplement: Supplementary file 1 [file ijms-22-05075-s001.zip › Figure 5.tif]
